# Do doctors appreciate that social isolation and loneliness are health issues?

**DOI:** 10.3389/fpubh.2024.1477228

**Published:** 2024-12-19

**Authors:** Brian Lawlor, Roger O'Sullivan, Gerry Leavey, Jim Lubben

**Affiliations:** ^1^The Global Brain Health Institute, Trinity College Dublin, Dublin, Ireland; ^2^Ageing Research and Development Division, The Institute of Public Health, Dublin, Ireland; ^3^The Bamford Centre for Mental Health and Well Being, Ulster University, Coleraine, United Kingdom; ^4^School of Social Work, Boston College, Chestnut Hill, MA, United States; ^5^Luskin School of Public Affairs, University of California, Los Angeles, Los Angeles, CA, United States

**Keywords:** loneliness, health, risk, care, training, social isolation

In 2023, The Lancet published an editorial entitled “Loneliness as a health issue,” which underscored the complexity of addressing loneliness and emphasized the key role that health professionals can play (1). Reports from the US Surgeon General (2) and the National Academies of Sciences, Engineering, and Medicine (3) also stress the importance of mobilizing the health sector, recognizing that healthcare professionals have a crucial position in addressing loneliness and social isolation, particularly due to their interaction with high-risk populations. Despite an increased awareness of social isolation and loneliness during the COVID pandemic, it remains uncertain whether the healthcare community fully recognize it as a significant health risk (4, 5). Historically, a patient's level of social connections was considered a personal matter (6), yet since the 1980s, there has been growing recognition of the impact of social connections on health. In House's seminal paper published in Science in 1988, the authors provided evidence that the quality of social relationships has the same impact on health and mortality as cigarette smoking and other major biomedical and psychosocial risk factors (7). Over the past 40 years there has been increasing siloing and demarcation between the health and social care systems and professions. This separation has not served patients well and may also have contributed to why medical specialties see loneliness as more of a social issue. However, loneliness and social isolation are important risk factors for all-cause mortality, stroke, heart disease, depression, suicide and dementia (8–10). Both are also key contributing factors and potentially treatable aspects of multimorbidity and the geriatric giants of cognitive impairment and frailty (2, 3, 11).

Doctors, for example, whether in primary, secondary or tertiary care, have the potential to play a key position in identifying lonely patients who need support. However, while concerns have been raised about the potential medicalization of loneliness (12), the reality is that this topic receives insufficient attention by doctors in their training or in their practice (4). So why is it that the medical field in general has been slow to acknowledge the importance of loneliness and social isolation for health and to act on this stark message? There are several possible explanations: the first of which is the stigma that is associated with social isolation and loneliness. Few people will ever admit to feeling lonely. Loneliness is often considered as a personal failure or weakness. For some clinicians, loneliness may be regarded as a natural aspect of aging and loss. Additionally, loneliness may be regarded as part of being human, or an emotion rather than a treatable disorder or condition. However, like depression, loneliness can have different levels of severity, duration and quality and we now know that chronic and persistent loneliness and social isolation negatively impacts quality of life, functioning and many and major health outcomes (13). Social isolation and loneliness can be confused and while they often overlap and co-occur, they can exist separately, and both should be measured to evaluate health risks and potential interventions (14–16). Some would argue that issues around definition and measurement of these social constructs have stalled the incorporation of loneliness and social isolation into mainstream medicine (17, 18). The assessment of other biomedical risk factors such as hypertension, obesity and smoking are easier to quantify, and the subjective nature of loneliness harder to capture. While this is a challenge, and the overlap with social isolation and its measurement is a reality, the compelling nature of the growing evidence of the risk of loneliness and social isolation to health, even with these measurement caveats, should override this argument (13, 17–19).

It is imperative that mainstream medicine and geriatrics regard social isolation and loneliness as important risks to health and include them as part of comprehensive medical risk assessments and mainstream medical practice (20). We need much more than the recommendation of establishing a connection with the patient (1). Empathic connection is key but a cultural shift that involves a systematic approach embedded in both training and practice that becomes as natural as checking one's weight and blood pressure is also required (10). We must go beyond a medical social history that simply consists of “do you smoke, drink, live alone or are you married” to one that includes a more nuanced exploration of the quality and quantity of the person's social relationships, how satisfied they are with their social engagement and clarity around whether the person feels lonely or not. This level of medical social history taking and assessment is the first step toward a comprehensive plan to tackle the health risks of loneliness and social isolation.
